# The Insular Subregions Topological Characteristics of Patients With Bipolar Depressive Disorder

**DOI:** 10.3389/fpsyt.2020.00253

**Published:** 2020-04-15

**Authors:** Meihui Qiu, Geya Liu, Huifeng Zhang, Yueqi Huang, Shihui Ying, Jinhong Wang, Ting Shen, Daihui Peng

**Affiliations:** ^1^ Division of Mood Disorders, Shanghai Mental Health Center, Shanghai Jiao Tong University School of Medicine, Shanghai, China; ^2^ Department of Medical Psychology, Xinhua Hospital Affiliated to Shanghai Jiao Tong University School of Medicine, Shanghai, China; ^3^ Shanghai Pudong New Area Mental Health Center, Tongji University School of Medicine, Shanghai, China; ^4^ Institute of Biomedical Engineering, School of Communication and Information Engineering, Shanghai University, Shanghai, China; ^5^ Department of Medical Imaging, Shanghai Mental Health Center, Shanghai Jiao Tong University School of Medicine, Shanghai, China; ^6^ Department of Psychiatry, Shanghai Mental Health Center, Shanghai Jiao Tong University School of Medicine, Shanghai, China

**Keywords:** bipolar disorder, functional connectivity, resting-state magnetic resonance imaging, insular subregions, neural substrate

## Abstract

The insular cortex appears to have a crucial role in emotional processing and cognitive control in bipolar disorder (BD). However, most previous studies focused on the entire insular region of BD, neglecting the topological profile of its subregions. Our study aimed to investigate its subregion topological characteristics using the resting-state functional connectivity (rsFC) in patients with BD on depression episode. The magnetic resonance imaging (MRI) data of 28 depressed BD patients and 28 age- and gender-matched healthy controls (HCs) were acquired. We observed that compared to HCs, depressed patients with BD exhibited significantly decreased rsFC between the right ventral anterior insula (vAI) and the left middle temporal gyrus/the right angular, the right dorsal anterior insula (dAI) and the left precuneus, as well as the right posterior insula and the right lingual gyrus. Furthermore, hyperconnectivity was observed between the left dAI and the left medial frontal gyrus, as well as right dAI and left superior temporal gyrus in BD depression. However, no significant group effect was observed between aberrant FC patterns and clinical variables. These findings revealed the functional connectivity patterns of insular subregions for the depressed BD patients, suggesting the potential neural substrate of insular subregions involved in depressive episode of BD. Hence, these results may provide a neural substrate for the potential treatment target of BD on depression episode.

## Introduction

Bipolar disorder (BD) is a chronic mental disease, characterized by alternating episodes between mania and depression (1). The high frequency and long duration of depressed symptoms is susceptible to psychosocial dysfunction and poor treatment, which further increase disease's burden, even the risk of suicide (2, 3). Therefore, it is a compelling need to investigate the mechanisms of depression in BD.

Accumulating evidence from neuroimaging studies have suggested that the insular cortex is critically involved in the pathogenesis of BD (4–6). Recent meta-analyses of studies using voxel-based morphometry revealed that patients with BD had aberrant structure and morphology in the insula (7, 8). Furthermore, one positron emission tomography (PET) study found that BD patients showed significantly higher binding rate of serotonin transporter in the insula (9).

Interestingly, the insula consists of several subregions, namely the ventral anterior insula (vAI), the dorsal anterior insula (dAI), and posterior insula (PI) (10), showing distinct histological characteristics (11). Meanwhile, this segmentation of insular cortex was confirmed by diffusion tensor imaging (DTI) data (12, 13). Previous studies found decreased volume of bilateral AI in BD patients (14, 15), which might be associated with abnormal emotional regulation in BD (16, 17). Besides, AI may provide transdiagnostic signatures to differentiate BD from major depressive disorder (MDD) (18). Taken together, the insular subregions may play distinctive roles on the pathogenesis of BD. Therefore, it will be meaningful to explore the topological profiles of insular subregions in BD.

On the other hand, the functional connectivity (FC) has been successfully applied for mapping complex neural circuits, reflecting the organization of brain networks. Numerous functional magnetic resonance imaging (fMRI) studies have demonstrated that BD patients showed abnormal FC patterns in some specific brain regions, such as between the pregenual anterior cingulate cortex (ACC) and amygdala/thalamus/pallidostriatum, respectively (19), as well as between amygdala and dorsal lateral prefrontal cortex (VLPFC) (20). Notably, the aberrant FC pattern between the insula and the PFC has also been observed in BD patients (21). Besides, previous study revealed that the aberrant FC between the AI and the inferior parietal lobule (IPL) of the executive control network (ECN) contributed to distinguishing dimension of emotion regulation between BD and MDD. Thus, the distinct FC patterns of insular subregions may provide potential neural substrate underlying emotion regulation dimension in BD patients.

In this study, we examined intrinsic FC of insular subregion in patients with BD. We hypothesized that depressed patients with BD would exhibit disrupted FC between insular subregions and some specific brain regions associated with emotion regulation. Furthermore, we hope to explore the relationship between the aberrant FC patterns of insular subregions and the symptom dimensions of BD on the episode.

## Methods

### Participants

Twenty-eight patients with BD on depression episode were enrolled from outpatient departments at Shanghai Mental Health Center. The BD patients were diagnosed independently by two physicians based on the Structured Clinical Interviews for Diagnostic and Statistical Manual Fourth Edition (DSM-IV). Including criteria: having been diagnosed as BD with current depression episode, aged 18–60 years, being right-handed, and having more than 9 years of education. To reduce the risk of mood instability, participants with BD were allowed to continue medication treatment, such as lithium, atypical antipsychotics, anticonvulsants (e.g., valproate, lamotrigine, carbamazepine, or topiramate), and antidepressants. The 24-item Hamilton Rating Scale for Depression (HAMD) >20 (22) and the Young Mania Rating Scale (YMRS) <7 (23) were collected to assess the clinical symptoms of BD patients. Thirty age- and gender-matched healthy volunteers were recruited from local community by advertisement. Excluding criteria of all participants were as follows: having a history of Axis I or Axis II psychiatric disorders of DSM-IV, having a history of substance dependence or substance abuse within the 6 months prior to assessment, having a history of electroconvulsive therapy, suffering serious neurological or medical disorders (e.g., head trauma and epilepsy), and other MRI contraindications (e.g., pregnancy and breast-feeding).

The study was approved by the Investigational Review Board (IRB00002733—Shanghai Mental Health Center, China). All participants gave written informed consent after a full description of the aims and design of the study.

### Image Acquisition

MRI raw data was acquired using Siemens 3.0 T MRI scanner in Shanghai Mental Health Center. High-resolution T1 images were acquired by the gradient recalled echo (GRE) sequence as the following parameters: repetition time (TR) = 2300 ms, echo time (TE) = 2.96 ms, field of view (FOV) = 24 × 24 cm^2^, slice thickness = 1.0 mm, 192 slices, gap = 0.0 mm, voxel = 1.0 × 1.0 × 1.0 mm^3^, matrix = 240 × 256, and scanning time = 9 min 14 s. Resting-state images were collected by echo planar imaging (EPI) sequence as the following parameters: TR/TE = 2000 ms/30 ms, FOV = 220 × 220 mm^2^, slice thickness = 4.0 mm, 33 slices, gap = 0.6 mm, voxel = 3.4×3.4×4.0 mm^3^, scanning time = 6 min 46 s, and 200 bolds. During the scanning, the participants were instructed to keep resting with their eyes closed.

### Data Preprocessing

Resting-state fMRI images were preprocessed using a toolbox of Data Processing and Analysis for Brain Imaging (DPABI, http://rfmri.org/dpabi). The first 10 volumes from each subject were discarded for the stability of the initial magnetic resonance imaging signal. For each participant, fMRI scans were first realigned to correct for head motion. Exclusion criteria for excessive head motion were >2.5 mm and/or translation >2.5° rotation. The nuisance covariates (i.e., the six motion parameters, the first time derivations, signals of the global brain, cerebrospinal fluid, and white matter) were regressed out from the MRI data. The processed data were band-pass filtered by using a frequency range of 0.01–0.08 Hz. A two-step coregistration method were used to transform the regressed fMRI data into the Montreal Neurological Institute (MNI) space: first, each subject's structural images were coregistered with the mean realigned fMRI image; then the structural images were segmented into gray matter, white matter, and cerebrospinal fluid on the basis of transformation parameters that coregistered with the MNI T1-weighted template. Realigned images were then normalized to the MNI space and resampled to 3 × 3 × 3 mm^3^ voxels. Finally, the images were smoothed with an 8-mm full width at half maximum (FWHM) Gaussian kernel. We also calculated frame-wise displacement (FD), which indexes the volume-to-volume changes in head position (24). There was no significant difference in mean FD (*T* = 0.02, *p* = 0.87) between BD patients (0.15 ± 0.09) and healthy controls (HCs; 0.16 ± 0.08).

### Definition of Insular Subregions

The insular seed regions of interest (ROI) were defined using masks based on the previous study (25). Selection of target ROIs of the insular subregions were defined using max voxel locations as described in Deen et al. (25). Spherical ROI masks (3 mm diameter) were created for each of the target ROIs using the DPABI, with max voxel locations as reported in Deen et al. specified as center of sphere (**Table 1**).

**Table 1:** The MNI coordinates of the ROIs.

	The MNI coordinates		
	X	Y	Z
L_vAI	−33	13	−7
R_vAI	32	10	−6
L_dAI	−38	6	2
R_dAI	35	7	3
L_PI	−38	−6	5
R_PI	35	−11	6

L, left; R, right; vAI, ventral anterior insula; dAI, dorsal anterior insula; PI, posterior insula; MNI, Montreal Neurological Institute; ROIs, regions of interest.

### Resting-State fMRI Analyses

Connectivity maps were obtained at the individual subject level for bilateral subregions within the insular seed regions by averaging the signal across all voxels in the ROI. Then, to calculate Pearson's correlation between the mean ROI time-series and the time-series from each whole brain acquired voxel. Correlation maps were converted to z-maps using Fisher's r-to-z transformation. Mean Fisher's z transformed values were extracted from target ROI masks using MarsBar and imported into SPSS (IBM, version 19.0) for analysis.

### Seed-to-Voxel

A whole-brain approach was used to explore whole-brain FC anchored on bilateral insular subregions in BD and HCs. Seed-to-voxel analyses of the FC differences between the groups were performed separately using the two-sample *t*-test using DPABI, as the age, gender, education, and mean FD were covariates. The significant threshold was *p* < 0.05, and it was corrected for multiple comparisons with a Gaussian random field (GRF) correction. Once a significant FC difference between patients and controls (*p* < 0.05, voxel z value > 2.3, GRF corrected) was observed, multiple comparison corrections were performed to identify the surviving clusters.

### Relationship Analysis Between Clinical Variables and FC Patterns

We extracted the mean values of significantly aberrant FC patterns. Then, Pearson's partial correlations (two-tailed) were conducted between significantly aberrant FC and HAMD scores, controlling for age and gender. Notably, the HAMD scale was categorized into seven subscale factors based on its Chinese version, including anxiety/somatization, change of weight, cognitive dysfunction, atypical circadian rhythm, retardation, sleep disorder, and desperation.

## Results

### Demographics and Clinical Characteristics

No significant differences were observed in gender (male/female: 14/14 vs. 18/10), age (31.79 ± 12.83 vs. 33.79 ± 9.95), and education (13.32 ± 3.37 vs. 13.64 ± 3.35) between BD patients and HCs (all *p* > 0.05). The detailed information was showed in **Table 2**.

**Table 2:** Demographic and clinical characteristics of BD and HCs groups.

	BD (n = 28)	HCs (n = 28)	*T*/χ^2^	*p*
Gender (M/F)	14/14	18/10	1.17^ǂ^	0.28
Age (years)	31.79 ± 12.83	33.79 ± 9.95	0.65^ǂǂ^	0.52
Education (years)	13.32 ± 3.37	13.64 ± 3.35	0.36^ǂǂ^	0.72
HAMD score	31.04 ± 7.92	–	–	–
Psychotropic medications, no.	23	–	–	–
Antidepressants	9	–	–	–
Lithium	7	–	–	–
Antiepileptic	10	–	–	–
Anxiolytics	4	–	–	–
Antipsychotics	13	–	–	–
Medication-free, no.	5	–	–	–

ǂ Chi-square test for the gender distribution between BD and HCs groups.

ǂǂ Two-sample t-test for the group differences in both age and education.

BD, bipolar disorder; HCs, healthy controls; HAMD, Hamilton Depression Rating Scale; M, male; F, female.

### Group Differences in Seed-Based Insular-Subregion Networks

**Figure 1:**
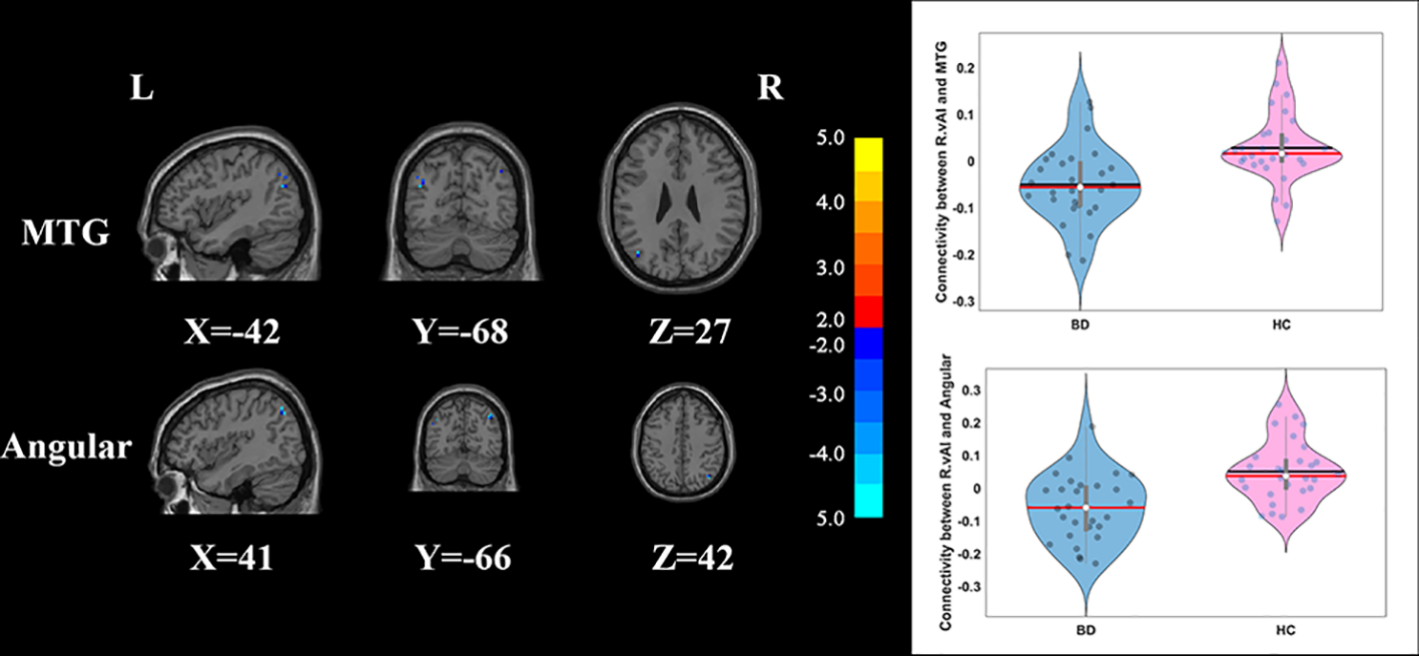
Group differences of the whole-brain functional connectivity anchored in R_vAI: compared to HCs, BD patients showed significantly decreased functional connectivity between R_vAI and MTG, as well as R_vAI and angular (GRF corrected, *p* < 0.05, voxel *Z* value > 2.3). Blue indicates smaller values in BD. L, left; R, right; BD, bipolar disorder; HC, healthy control; R_vAI, right ventral anterior insula; MTG, middle temporal gyrus; GRF, Gaussian random field.

**Figure 2:**
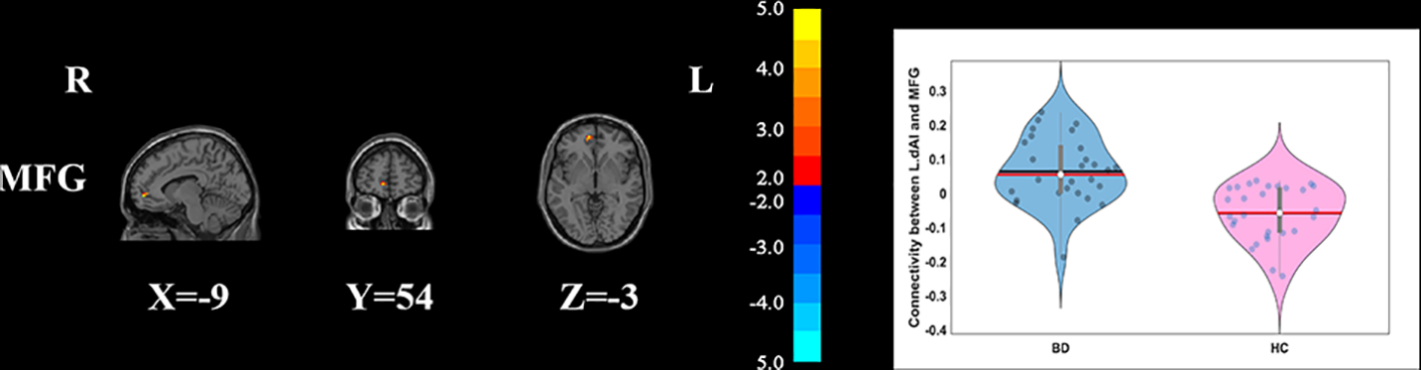
Group differences of the whole-brain functional connectivity anchored in L_dAI: compared to HCs, BD patients had significantly higher functional connectivity between L_dAI and MFG (GRF corrected, *p* < 0.05, voxel *Z* value > 2.3). Red indicates larger values in BD. L, left; R, right; BD, bipolar disorder; HC, healthy control; L_dAI, left dorsal anterior insula; MFG, middle frontal gyrus; GRF, Gaussian random field.

**Figure 3:**
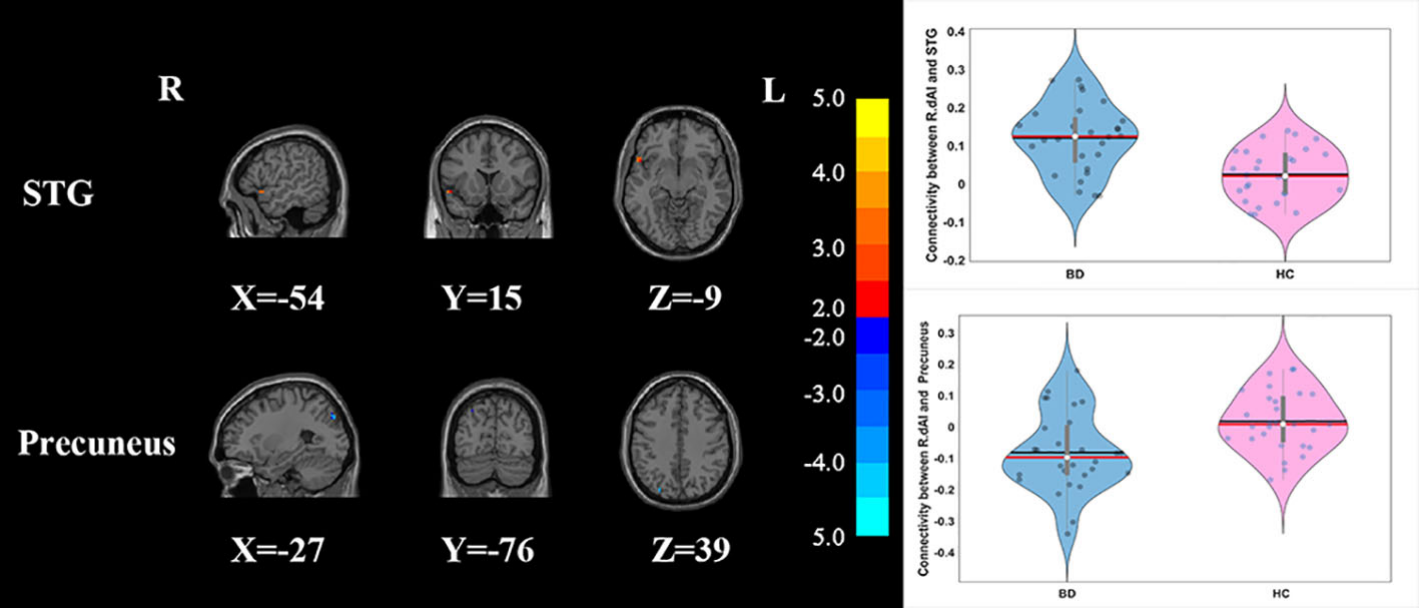
Group differences of the whole-brain functional connectivity anchored in R_dAI: while showing decreased functional connectivity between R_dAI and precuneus, BD patients had significantly higher functional connectivity between R_dAI and STG compared to HCs (GRF corrected, *p* < 0.05, voxel *Z* value > 2.3). Blue indicates smaller values in BD and red indicates larger values in BD. L, left; R, right; BD, bipolar disorder; HC, healthy control; R_dAI, right dorsal anterior insula; STG, superior temporal gyrus; GRF, Gaussian random field.

**Figure 4:**
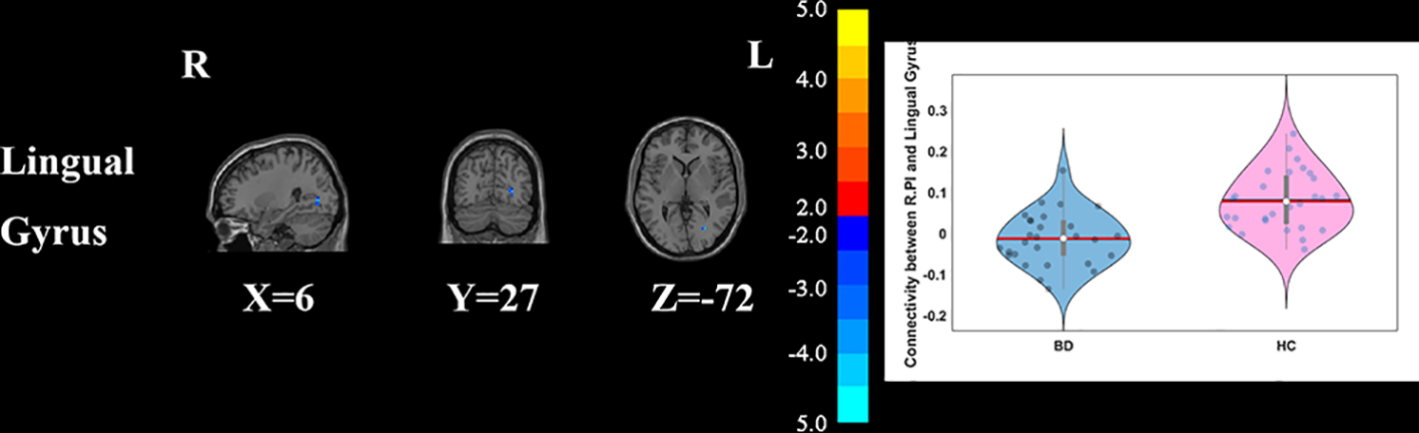
Group differences of the whole brain functional connectivity anchored in R_PI: BD patients had significantly decreased functional connectivity between R_PI and lingual gyrus (GRF corrected, *p* < 0.05, voxel *Z* value > 2.3). Blue indicates smaller values in BD. L, left; R, right; BD, bipolar disorder; HC, healthy control; R_PI, right posterior insula; GRF, Gaussian random field.

Compared with HCs, the patients with BD had significantly decreased FC between the R_vAI and the left middle temporal gyrus (*T* = −4.66, *p* < 0.05, **Figure 1**), as well as the right angular (*T* = −5.17, *p* < 0.05, **Figure 1**). BD patients had increased FC than HCs between the L_dAI and the left medial frontal gyrus (MFG; *T* = 5.51, *p* < 0.05, **Figure 2**). While showing increased FC between the R_dAI and the left superior temporal gyrus (STG; *T* = 4.19, *p* < 0.05, **Figure 3**), BD patients had significantly decreased connectivity between the R_dAI and the left precuneus (*T* = −4.88, *p* < 0.05, **Figure 3**) than HCs. Patients with BD also had significantly decreased connectivity than HCs between the R_PI and the right lingual gyrus (*T* = −4.41, *p* < 0.05, **Figure 4**). The detailed information was observed in **Table 3**. These results survived even after correction for multiple comparisons (*p* < 0.05, voxel z value > 2.3, GRF corrected). No other group differences were observed by seeding the L_vAI or L_PI (*p* > 0.05, voxel z value > 2.3, GRF corrected).

**Table 3:** Group differences in seed-based functional connectivity of the insular subregions.

Seed	Connected regions	L/R	Voxels	BA	MNI coordinates			*T*
					X	Y	Z	
R_vAI	Middle temporal gyrus	L	14	39	−42	−68	27	−4.66
	Angular	R	7	19	41	−66	42	−5.17
L_dAI	Medial frontal gyrus	L	12	29	−9	54	−3	5.51
R_dAI	Superior temporal gyrus	L	8	31	−54	15	−9	4.19
	Precuneus	L	7	6	−27	−76	39	−4.88
R_PI	Lingual gyrus	R	45	6	27	−72	3	−4.41

BA, Broadmann area; BD, bipolar disorder; HC, healthy controls; dAI, dorsal anterior insula; AI, ventral anterior insula; PI, posterior insula; MNI, Montreal Neurological Institute.

### Associations Between Insular Subregions Connectivity and Clinical Symptoms

We explored the relationships between these abnormal FC patterns of the insular subregions and clinical symptoms. However, no significant correlation was found between FC indexes and age, depression, or other clinical characteristics within BD group.

## Discussion

Using a seed-based ROI analyses, our study showed the aberrant FC between right vAI and left middle temporal gyrus, right vAI and right angular, left dAI and left MFG, right dAI and left STG, right dAI and left precunus, as well as right PI and lingula gyrus. Therefore, the present study provides evidence that the insular subregions have aberrant FC patterns in BD patients on depression episode.

Emerging evidence suggests that the insular cortex, as an integral hub of salience network (SN), plays a pivotal role in behavioral stimuli detection modulating the dynamic coordination between internal and extra-personal stimuli, and integrating information of diverse cognitive control, emotional processes (26–29). Previous findings showed that BD was associated with abnormal structure and function in specific subdivisions of the insula (30–32). Neuroimaging studies focusing on the resting-state FC (rsFC) of insular subdivisions revealed the discriminative ability of dysfunctional connectivity patterns of anterior insula for bipolar depression (18, 33). Hence, the aberrant profiles of insular subregions may provide a novel insight for the pathophysiology of BD depression.

Our finding showed increased FC between right AI and several specific brain regions, including the middle temporal gyrus and angular, which are known as nodes of the default network (DMN). These findings are consistent with previous studies of aberrant FC in BD (33–35). Ellard et al. (33) observed that compared to patients with unipolar depression and HCs, BD patients showed significantly aberrant FC between right AI and the IPL in DMN. The DMN might involve in self-referential mental process and social cognition (29, 31, 36, 37). Furthermore, it is reported that the DMN was associated with the symptom of BD patients, such as rumination. Lois and his colleague found decreased FC within the DMN in remitted BD patients (38). Converging evidence from neuroimaging studies using memory tasks indicated that the DMN involves in the retrieval processing of self-related episodic memory (39, 40). In consistent with previous findings, our findings revealed that abnormal intra-network between the SN and the DMN involved in BD on the depression episode (5).

Among insular subregions, the vAI is closest to limbic cortex showing extensive relationships with other cortical regions, while the dAI primarily is connected with dorsal ACC (dACC) along with other regions of control networks(41–43). Consistently, our results demonstrated increased FC between left vAI and MFG, STG, and precuneus in BD depression. Furthermore, our study observed that depressed patients with BD had aberrant rsFC profiles anchored on dAI, including the hyperconnectivity with the MFG and the STG, and hypoconnectivity with the precuneus. The MFG and STG has been identified as key nodes in ECN involvement in goal-directed behavior and cognitive control (26, 44, 45). Previous studies have found the altered FC between dAI and the IPL in the ECN, which was related to impairments of perceived emotion control (33). Additionally, our study observed that BD patients had hypoconnectivity between dAI and precuneus. As a key node of DMN, the precuneus is important for self-reference processing (46), consciousness (47), integration of past and present information (48), and perspectives of social interaction (49). Young and his colleague found that BD patients showed increased hemodynamic activity in the anterior insula during positive memories recall of specific autobiographical memory (AM) tasks, while showing decreased activity in the precuneus during negative memories recall of AM tasks (50).

As a major hub of the SN, the AI serves as identifying the salient stimuli information and forwarding to higher cognitive regions (31). Furthermore, emerging evidence supports the idea that the AI might perceive regulatory control demands and facilitate dynamic switching between DMN and ECN (28, 29, 51). Interestingly, our results showed aberrant rsFC patterns of the AI, including hyperconnectivity with nodes of ECN and hypoconnectivity with nodes of DMN. These results indicated that the AI could integrate the abnormal affective and cognitive process in BD patients, and facilitate the switching between DMN and ECN (28, 29, 51).

As for the rsFC patterns of PI, we detected its dysconnectivity with the lingual gyrus within the visual recognition network, which may be involved in the perception of facial emotion stimuli (52–54). Neuroimaging studies using rsFC and DTI approaches have observed the abnormality of lingual gyrus in patients with BD (55, 56). Consistently, numerous task-based fMRI studies found abnormal activation of lingual gyrus in patients with BD during emotional face processing (57).

## Limitation

Although our study provided substantial evidences showing abnormal FC between insular subregions and other brain areas, several limitations should be considered when interpreting our findings. First, our study reveals the potential mechanism of insular subregions' connectivity patterns underlying BD. However, the cross-sectional study may neglect the characterization of disease's development trajectory, and ignore the dynamic changes of brain function along with mental states. Second, the sample size of our study is modest, which may impose some restriction on the reliability and generality of our findings. Third, although we acknowledge the well-established relationship between abnormal rsFC patterns of insular subregions and clinical symptoms, we failed to replicate the significant correlation in our study. It may be due to the less sensitivity of HAMD scale for its variety of clinical symptoms in BD patients on depression episode. And lastly, most patients with BD were treated with lithium, antiepileptics, anxiolytics, or antidepressants, and even some with frequent polymedication at the time of MRI in the study. We further explored the possible effects of BD medication on insular subregions connectivity, and finally found an effect of antiepileptic on lingual regions (in the file of Supporting Information-2). As a consequence, further research is needed to assess the effects of psychotropic medications on BOLD signal with a relatively large sample to replicate our results in future study.

## Conclusion

Our study found that BD patients on depression episode had abnormal FC among insular subregions and other brain regions, including the medial temporal gyrus, angular, MFG, STG, precuneus, and lingual gyrus involved in DMN, ECN, and the visual recognition network. Considering that these regions related to the emotional process and cognitive control, our findings provided substantial evidence of abnormal brain functional network of BD on depression episode.

## Data Availability Statement

The datasets generated for this study are available on request to the corresponding authors.

## Ethics Statement

The studies involving human participants were reviewed and approved by Shanghai Mental Health Center. The patients/participants provided their written informed consent to participate in this study.

## Author Contributions 

DP and TS designed and supervised the project. MQ, GL, HZ, and YH were responsible for the collection of participants. MQ and GL undertook the analysis of raw MRI data and the preparation of the manuscript. JW and SY gave the guide of the data analysis. All authors have participated in the revision of the finial manuscripts

## Funding

This work was supported by the grant from the National Natural Science Foundation of China (Grant No. 81971269), cross-disciplinary and translational medical research of Shanghai Jiao Tong University (Grant No. ZH2018ZDA29), and Key Clinical Research Program of Shanghai Mental Health Center (Grant No. CRC2018ZD05).

## Conflict of Interest

The authors declare that the research was conducted in the absence of any commercial or financial relationships that could be construed as a potential conflict of interest.
