# Supplementary material for: The Insular Subregions Topological Characteristics of Patients With Bipolar Depressive Disorder
Source: Front Psychiatry. 2020 Apr 15;11:253. doi: 10.3389/fpsyt.2020.00253 (PMC7175992; doi:10.3389/fpsyt.2020.00253)
Supplement: Supplementary file 1 [file DataSheet_1.docx]

**Supporting information: Functional Connectivity Analysis**

**Methods:**

**ROI-to-ROI analysis among insular subregions**

To explore which of insula subregions were contributing to the abnormal connectivity with the resulting clusters, ROI-to-ROI analyses were performed using the insula subregions as seed ROIs. The insula was divided into six subregions: left/ right ventral anterior (vAI), left/right dorsal anterior (dAI), and left/right posterior (PI). ROIs 3mm radius spheres centered at MNI coordinates reported previously [1] that were created using the WFU-pickatlas (http://fmri.wfubmc.edu/software/pickatlas). DPABI toolbox was adopted to compute correlations between the BOLD signals of all six ROIs. Fisher’s Z-transformed correlations were calculated to indicate the strength of connectivity between each pairs of ROIs. Group effect between BD and HCs was measured using Network-based statistic toolbox (https://www.nitrc.org/projects/nbs/). Specifically, significant group difference were reported if p < 0.05 using false discovery rate (FDR) correction at nonparametric permutation test (50000 permutations).

**Results:**

**ROI-to-ROI analysis among insular subregions**

Compared to HCs group, BD group showed significantly attenuated rsFC between the left dAI and the left PI (*t* = 3.19, p < 0.05) . However, no other significant connections were observed between BD and HCs.


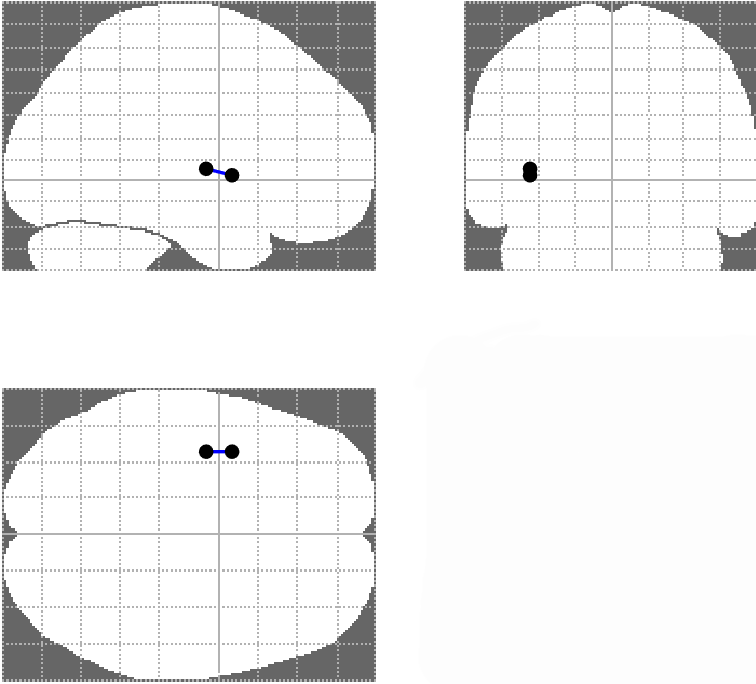


Figure S1 Significant functional connectivity in left dAI and the left PI between BD and HCs

Abbreviation: dAI, dorsal anterior insular; PI, posterior insular; BD, bipolar disorder; HCs, healthy controls.

**Discussion**

The insula, as a multimodal integration region, evaluates emotional or motivational salience of certain external and internal stimulus [2]. Specifically, the dAI is functionally connected with the entire insula, and primarily connected with dorsal anterior cingulate cortex (dACC) along with other regions of control networks. Furthermore, the PI is highly connected with the entire insula, opercula, dorsal ACC, IFG, and the primary and secondary motor and somatosensory cortices. Thus, it has been proposed that the dAI and PI plays critical roles in modulating and processing affective aversive stimuli [1,4].

According to the supplementary the ROI-to-ROI analysis, our findings revealed that BD patients showed attenuated connection between the left dAI and the left PI. Thus, combining with the seed-to-voxel analyses, we hypothesized that attenuated left dAI-PI connection within insula might the primary physiological aberration[5-6], though the cross sectional design may precludes the cause and effect. Consistently, previous studies suggested that AI hypometabolism was associated with poor response to antidepressant treatment, but showing remission after psychotherapy in depressed patients[7]. Hence, future cohort studies, or experimental rTMS intervention, should focus on the dAI-PI connection as a suitable target biomarker for further understanding the pathogenesis of BD, as well as possible potential intervention and physical therapy.

**References**

1. Deen B, Pitskel NB, Pelphrey KA. Three systems of insular functional connectivity identified with cluster analysis. Cereb Cortex. 2011;21:1498–1506.
2. Damasio A, Damasio H, Tranel D. Persistence of feelings and sentience after bilateral damage of the insula. Cereb Cortex. 2013;23:833–846.
3. Chang LJ, Yarkoni T, Khaw MW, Sanfey AG. Decoding the role of the insula in human cognition: Functional parcellation and large-scale reverse inference. Cereb Cortex. 2013;23:739–749.
4. Dosenbach NUF, Fair DA, Miezin FM, Cohen AL, Wenger KK, Dosenbach RAT, Fox MD, Snyder AZ, Vincent JL, Raichle ME, et al. Distinct brain networks for adaptive and stable task control in humans. Proc Natl Acad Sci U S A. 2007;104(26):11073–11078.
5. Christoff K, Gabrieli JDE. The frontopolar cortex and human cognition: Evidence for a rostrocaudal hierarchical organization within the human prefrontal cortex. Psychobiology. 2000;28:168–186.
6. Ambrosi E, Arciniegas DB, Madan A, et al. Insula and amygdala resting-state functional connectivity differentiate bipolar from unipolar depression. Acta Psychiatr Scand. 2017;136(1):129–139.
7. McGrath CL, Kelley ME, Holtzheimer PE, III, et al. Toward a Neuroimaging Treatment Selection Biomarker for Major Depressive Disorder. Jama Psychiatry. 2013;70:821–829
