# Supplementary material for: The Insular Subregions Topological Characteristics of Patients With Bipolar Depressive Disorder
Source: Front Psychiatry. 2020 Apr 15;11:253. doi: 10.3389/fpsyt.2020.00253 (PMC7175992; doi:10.3389/fpsyt.2020.00253)
Supplement: Supplementary file 2 [file DataSheet_2.docx]

**Supplementary Analyses**

Medication status among BD patients were categorized into two groups, namely using medication and medication naïve. Spearman correlation analysis were adopted to explore the medication status (use/no use) and FC values in BD patients using SPSS (IBM, version 19.0) with a statistical threshold of *p*<0.05. However, no significance correlations were survived the statistical threshold.

Then, we further explore whether the different types of medication (e.g., antidepressant, mood stabilizer, and antiepileptics etc.) would influence the FC values. Statistical analyses were carried out with SPSS (IBM, version 19.0) to explore the association between different types of medication (the use of psychotropic medications, antipsychotic medication, and use of lithium, antiepileptics, anxiolytics, or antidepressants at the time of MRI), and the values of seed-based functional connectivity, using a statistical threshold of*p*<0.05. In our analysis of medication effects, we only found the use of antiepileptic medication at the time of MRI were associated with FC between the right posterior insula and lingual gyrus (Figuer. S2 and Table. S1), but not the use of antidepressant and mood stabilizer.


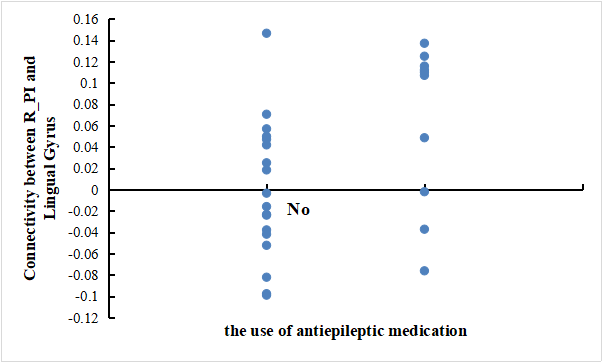


Figure S2. The significant correlation between connectivity between the right posterior insula and lingual Gyrus and the use of antiepileptic medication at the time of MRI within BD group. R: right, PI: posterior insula.

Table.S1: Exploratory correlation analysis of associations between current treatment of psychotropic medications and FC differences between the right posterior insula and lingual gyrus

| Current treatment | Lingual Gyrus | |
| --- | --- | --- |
|  | *ρ* | P |
| Antiepileptic [yes/no] | 0.415 | 0.028 |

Abbreviations: *ρ* Spearman’s rho

Table. S2: Antiepileptic medication usage among BD individuals.

| **BD Participant** | Antiepileptic **(daily dosage in mg)** |
| --- | --- |
| 1 | None |
| 2 | None |
| 3 | None |
| 4 | Valproate Sodium (500) |
| 5 | None |
| 6 | None |
| 7 | Magnesium Valproate (details for medication were missing) |
| 8 | None |
| 9 | None |
| 10 | Lamotrigine (25) |
| 11 | None |
| 12 | Valproate Sodium (750) |
| 13 | Lamotrigine (75) |
| 14 | None |
| 15 | Magnesium Valproate (750) |
| 16 | Valproate Sodium (500) |
| 17 | None |
| 18 | Lamotrigine(25) |
| 19 | None |
| 20 | None |
| 21 | None |
| 22 | Lamotrigine (details for medication were missing) |
| 23 | None |
| 24 | None |
| 25 | None |
| 26 | None |
| 27 | None |
| 28 | Magnesium Valproate (details for medication were missing) |
